# Supplementary material for: Little evidence that Eurasian jays protect their caches by responding to cues about a conspecific’s desire and visual perspective
Source: eLife. 2021 Sep 10;10:e69647. doi: 10.7554/eLife.69647 (PMC8536255; doi:10.7554/eLife.69647)
Supplement: Transparent reporting form [file elife-69647-transrepform1.pdf]

eLIFE
1st Floor
24 Hills Road
Cambridge CB2 1JP, UK
P 01223 855340
W elifesciences.org
T @elife

eLife's transparent reporting form

We encourage authors to provide detailed information *within their submission* to facilitate the interpretation and replication of experiments. Authors can upload supporting documentation to indicate the use of appropriate reporting guidelines for health-related research (see EQUATOR Network), life science research (see the BioSharing Information Resource), or the ARRIVE guidelines for reporting work involving animal research. Where applicable, authors should refer to any relevant reporting standards documents in this form.

If you have any questions, please consult our Journal Policies and/or contact us: editorial@elifesciences.org.

Sample-size estimation

- You should state whether an appropriate sample size was computed when the study was being designed
- You should state the statistical method of sample size computation and any required assumptions
- If no explicit power analysis was used, you should describe how you decided what sample (replicate) size (number) to use

Please outline where this information can be found within the submission (e.g., sections or figure legends), or explain why this information doesn't apply to your submission:

Because of the limited sample of captive jays in Clayton's Comparative Cognition Lab that could be tested, no power analysis was conducted to establish an appropriate sample size for the study. Instead, in each experiment, we used all the individuals that were available for testing at the time when the experiment was being conducted, out of all the Eurasian jays housed in the facility that were familiar with the general set-up of caching experiments (i.e., the jays that would cache in caching trays placed in the indoor testing compartments; see 'Statistical Analysis' subsection in Materials and Methods). Notably, our samples are comparable to those from previous studies in this area.

Replicates

- You should report how often each experiment was performed
- You should include a definition of biological versus technical replication
- The data obtained should be provided and sufficient information should be provided to indicate the number of independent biological and/or technical replicates
- If you encountered any outliers, you should describe how these were handled
- Criteria for exclusion/inclusion of data should be clearly stated
- High-throughput sequence data should be uploaded before submission, with a private link for reviewers provided (these are available from both GEO and ArrayExpress)

eLife Sciences Publications, Ltd is a limited liability non-profit non-stock corporation incorporated in the State of Delaware, USA, with company number 5030732, and is registered in the UK with company number FC030576 and branch number BR015634 at the address 1st Floor, 24 Hills Road, Cambridge CB2 1JP | August 2014

Please outline where this information can be found within the submission (e.g., sections or figure legends), or explain why this information doesn't apply to your submission:

Each experiment was conducted once (see Materials and Methods).

14 Eurasian jays (*Garrulus glandarius*) participated in the study. Our jays are housed at the University of Cambridge, and, as is typical in animal cognition research, are repeatedly tested throughout their lifetime. Since their arrival in the laboratory (some birds in 2006 or 2008), and before the present study was conducted (2017-2018), birds from our sample of jays have been involved in a number of caching experiments. In Table 1, we reported for each individual bird i) the previous experimental history with regard to the experiments involving identical or very similar experimental set-up as those used in the present study, and; ii) the specific experiment in which the bird participated during the present study.

Each bird in our sample is a biological replicate, and when we took repeated measurements from the same birds, these can be viewed as technical replicates.

Outliers (see Figures 1-4) were not excluded from statistical analysis.

Criteria for exclusion/inclusion are described in the 'Specific Procedures' subsection (see Materials and Methods).

No genetic sequencing was conducted in the study.

Statistical reporting

- Statistical analysis methods should be described and justified
- Raw data should be presented in figures whenever informative to do so (typically when N per group is less than 10)
- For each experiment, you should identify the statistical tests used, exact values of N, definitions of center, methods of multiple test correction, and dispersion and precision measures (e.g., mean, median, SD, SEM, confidence intervals; and, for the major substantive results, a measure of effect size (e.g., Pearson's r, Cohen's d)
- Report exact p-values wherever possible alongside the summary statistics and 95% confidence intervals. These should be reported for all key questions and not only when the p-value is less than 0.05.

Please outline where this information can be found within the submission (e.g., sections or figure legends), or explain why this information doesn't apply to your submission:

Statistical analysis methods are described and justified in the 'Statistical Analysis' subsection (see Materials and Methods).

All plots in the manuscript show individual data together with a boxplot (see Figures 1-5).

For all statistical tests that were computed, we reported: i) the specific test that was used; ii) the sample size; iii) the exact values of the test statistic and of p (see Results). Median values were also reported for some statistical tests (see Results).

Because of the small size of our dataset, we reported the raw, unstandardized effect sizes – the differences in cache number between conditions – and plotted all raw data. For the non-parametric statistics we use, the standardized effect sizes are a transformation of the p-value relative to the sample size, e.g., Pallant (2007), and we feel the unstandardized effect sizes along with good data visualizations are a clearer way of communicating the magnitude of any effects.

(For large datasets, or papers with a very large number of statistical tests, you may upload a single table file with tests, Ns, etc., with reference to sections in the manuscript.)

Group allocation

- Indicate how samples were allocated into experimental groups (in the case of clinical studies, please specify allocation to treatment method); if randomization was used, please also state if restricted randomization was applied
- Indicate if masking was used during group allocation, data collection and/or data analysis

Please outline where this information can be found within the submission (e.g., sections or figure legends), or explain why this information doesn't apply to your submission:

This study did not require the allocation of the sample into experimental groups. Due to the within-subject design, in all experiments, the same individuals were tested in different conditions. A description of the method used to establish the order in which individuals were tested in different conditions is reported in the 'Specific Procedures' subsection (see Materials and Methods).

Due to the procedures utilized, it was not possible for the experimenters to be naïve about the experimental condition during data collection. Note, however, that during trials (i.e., at the time that the subjects exhibited the behavior related to the dependent variable) the experimenters were not present in the testing compartments (see 'General Procedures', Materials and Methods).

The experimenters were not naïve to the condition during data analysis. (see 'Data Collection' subsection in Material and Methods)

eLife Sciences Publications, Ltd is a limited liability non-profit non-stock corporation incorporated in the State of Delaware, USA, with company number 5030732, and is registered in the UK with company number FC030576 and branch number BR015634 at the address 1st Floor, 24 Hills Road, Cambridge CB2 1JP | August 2014

Additional data files ("source data")

- We encourage you to upload relevant additional data files, such as numerical data that are represented as a graph in a figure, or as a summary table
- Where provided, these should be in the most useful format, and they can be uploaded as "Source data" files linked to a main figure or table
- Include model definition files including the full list of parameters used
- Include code used for data analysis (e.g., R, MatLab)
- Avoid stating that data files are "available upon request"

Please indicate the figures or tables for which source data files have been provided:

Data and analyses (scripts for statistical tests and plots) of all experiments are available at http://doi.org/10.5281/zenodo.4636561
